# Effect of lymph nodes count in node-positive gastric cancer

**DOI:** 10.7150/jca.30979

**Published:** 2019-09-07

**Authors:** Wenjie Zhang, Guangyan Zhangyuan, Jincheng Wang, Kangpeng Jin, Yang Liu, Fei Wang, Weiwei Yu, Haitian Zhang, Guoqiang Li, Decai Yu, Huihui Chen, Qingxiang Xu, Beicheng Sun

**Affiliations:** 1 Department of Hepatobiliary Surgery, The Affiliated Drum Tower Hospital of Nanjing University Medical School, Nanjing 210029, Jiangsu Province, P.R. China; 2 Department of Hepatobiliary Surgery of Drum Tower Clinical Medical College, Nanjing Medical University, Nanjing, China.; 3 Liver Transplantation Center, The First Affiliated Hospital of Nanjing Medical University, Nanjing, Jiangsu Province, P.R. China.

**Keywords:** nomograms, lymph nodes, gastric cancer

## Abstract

**Background**: The retrieved lymph node (LN) count has been confirmed as a prognostic indicator in various cancers. However, the correlation between LN counts and patient prognosis in gastric cancer with node-positive is not fully studied.

**Methods**: A total of 8475 patients undergoing gastrectomy in Surveillance, Epidemiology, and End Results Program (SEER)-registered gastric cancer were analyzed. Kaplan-Meier methods and multivariable Cox regression models were used to analyze long-term outcomes and risk factors. Moreover, nomograms including LN counts were established to predict overall survival (OS) and cancer-specific survival (CSS), and Harrell's concordance index (c-index) was adopted to evaluate prediction accuracy.

**Results**: Patients were stratified into 1-6, 7-14, and > 14 subgroups according to the optimal cutoff for retrieved LNs in terms of 5-year CSS. Further analysis indicated that higher LN counts were an independent predictor of longer survival in each N category. Nomograms on CSS and OS were established according to all significant factors, and c-indexes were 0.663 and 0.654 (P< 0.001), respectively.

**Conclusions**: These results indicated that the more the LNs retrieved, the better the survival would be. Nomograms incorporating LN counts can be recommended as practical models to provide more accurate prognostic information for GC patients.

## Introduction

Gastric cancer (GC) ranks fourth in frequency in the world and is globally the second leading cause of cancer-related death 1, 2. GC is the most common malignancy in Latin America and Asia, and its incidence is nearly 10-fold higher than in the US 3. According to the 7th edition of the AJCC TNM classification, the minimum number of retrieved LNs is not defined 4. Meanwhile, the number of metastatic LNs was validated as an independent prognostic factor after surgical resection 5, 6. However, whether more retrieved LNs can be linked to accurate staging is controversial. In addition, there is doubt regarding the recommended minimum retrieval of 15 LNs for GC 7. Some studies sought to investigate the optimal LNs retrieval cutoff in node-negative GC, but few studies have focused on node-positive patients in a large population 8.

The objective of this retrospective study was to assess the effect of retrieved LN counts on the long-term survival outcome in node-positive gastric patients, and to explore the optimal retrieved LNs cutoff value. In this study, we searched the Surveillance, Epidemiology, and End Results (SEER) population-based database and analyzed the clinicopathological characteristics and cancer-specific survival of these subgroups. We also used the X-tile program to determine the optimal cutoff.

## Methods

### Patient selection

Data were obtained from the Surveillance Epidemiology and End Results (SEER) Program of the United States National Cancer Institute. The current SEER database consists of 18 population-based cancer registries that represent approximately 26% of the population in the United States. SEER data contain no identifiers and are publicly available for studies of cancer-based epidemiology and survival analysis.

Inclusion criteria included the following: (1) patients were diagnosed from 2004 to 2012; (2) the site code was limited to stomach; (3) underwent surgical resection; (4) age > 18 years old; (5) histology code was limited to adenocarcinoma (8140/3, 8144/3, 8255/3, 8211/3, 8260/3,8263/3), mucinous adenocarcinoma (8480/3), and signet ring cell carcinoma (8490/3); (6) at least with one LN retrieval; (7) information on CSS and OS available. The primary endpoint of the study is 5-year CSS, which was calculated from the date of diagnosis to the date of cancer-specific death. Cancer-specific deaths were treated as events, and deaths from other causes were treated as censored observations. The median follow-up of patients was calculated from the date of diagnosis to the date of cancer-specific death.

This study was based on public data from the SEER database; we obtained permission to access research data files with the reference number 10504-Nov2014. The data did not include the use of human subjects or personal identifying information. Thus, no informed consent was required for this part of the study. The methods were carried out in accordance with the approved guidelines in this study. Ethical approval was obtained from the institutional review board of Nanjing Medical University.

### Identification of the optimal cutoff point of retrieved LNs

The retrieved LNs cutoff points were produced and analyzed using the X-tile program, which identified the cutoff with the minimum p values from log-rank χ2 statistics for the categorical LN counts in terms of survival.

### Statistical analysis

Categorical variables were summarized using frequency (%). A comparison of the categorical variables between LNs count subgroups was conducted using Pearson's χ2 test. Continuous variables were compared using the Mann-Whitney U test. Survival curves were generated using the Kaplan-Meier method; differences between the curves were analyzed by the log-rank test. Multivariable Cox proportional hazards regression models were used to assess potential risk factors for CSS. Cox stepwise regression analysis was also performed to determine predictive factors for gastric cancer prognosis, with a significance level of 0.05 for entering and 0.10 for removing the respective explanatory variables. Nomograms for possible prognostic factors associated with CSS and OS were established by R software, and the model performance for predicting outcome was evaluated by Harrell's concordance index (c-index), which is a measure of discrimination.

All statistical analyses were performed using the statistical software package SPSS for Windows, version 17 (SPSS Inc., Chicago, IL, USA). The results were considered statistically significant when a two-tailed test provided a P-value of less than 0.05.

## Results

### Patient Characteristics

We identified 8475 eligible patients with GC meeting the eligibility criteria in the SEER database, including 5404 male and 3071 female. All patients had at least one LN examined. There were 2738 patients with N1 stage, 2493 patients with N2 stage, 2252 patients with N3a stage, and 992 patients with N3b stage. Patient demographics and pathological features are summarized in Table 1. Patients had a higher rate of poor/ anaplastic grade tumors, a higher ratio of cardia and gastric antrum tumors, a higher proportion of adenocarcinoma and T3/4 tumor stage across all the N (+) patients (P< 0.001). The median number of LNs examined was 17.89 (range, 9-23). The median positive LN count was 7.19 (range, 2-10).

### Identification of minimum number of retrieved LNs in node-positive patients

**Figure 1:**
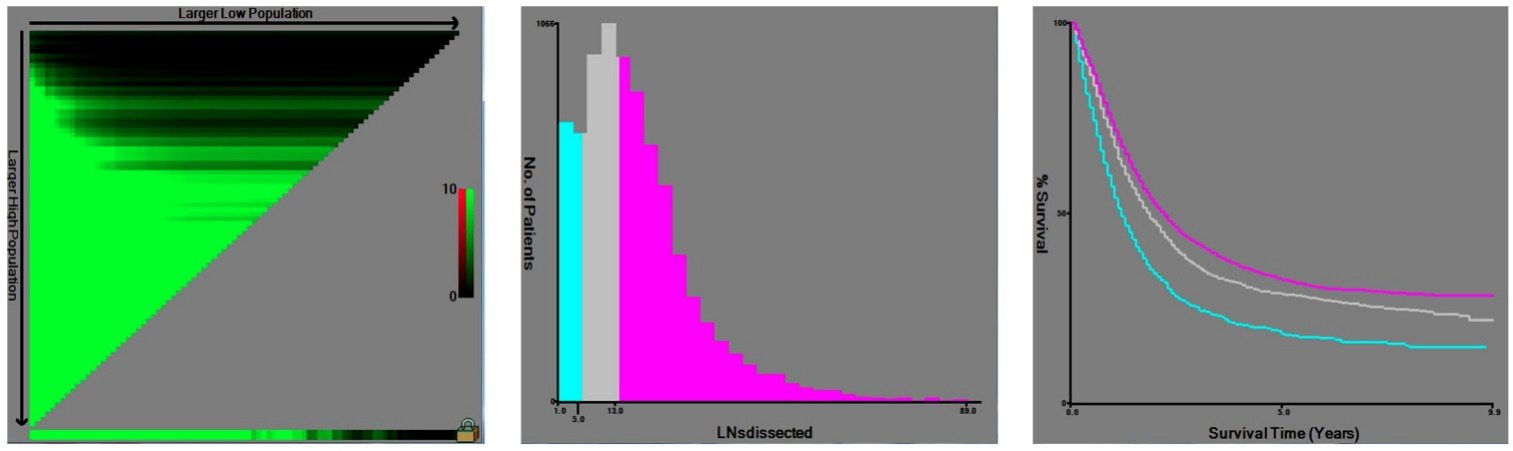
** X-tile analysis of survival data from the SEER registry.** X-tile analysis was done on patient data from the SEER registry, equally divided into training and validation sets. The optimal cut-point highlighted by the black circle in the left panels (A) is shown on a histogram of the entire cohort (middle panels) (B), and a Kaplan-Meier plot (right panels) (C). P values were determined by using the cut-point defined in the training set and applying it to the validation set. Figure 1 shows the optimal cutoff point for the lymph node positive patients (number 6 and 14, χ2=154.244, P < 0.001).

X-tile plots were constructed and the maximum of chi-square log-rank values of 154.244 (P< 0.001) was achieved when applying 6 and 14 as the cutoff value of retrieved LNs. This value can be used to divide the cohort into high, middle and low risk subsets in terms of gastric cancer-specific survival (GCSS), which were 20.3%, 29.0% and 32.6%, respectively (P< 0.001) (Fig. 1). Then, to investigate the impact of different LN counts on GCSS, we treated the number of LN counts as a continuous variable and analyzed the number of retrieved LNs from 2 to 20. The number of retrieved LNs was an independent prognosis factor for GC, and patients with 15 or more LNs retrieved had a relative14.4% improvement in 5-year GCSS compared to those with 6 less LNs retrieved (32.6% versus 18.2%). The 5-year GCSS of patients with N or more nodes increased gradually when N reaching 14. After the number 15, the survival rates were roughly stable between the compared groups (Table 2).

### Effect of LN counts on GCSS rates in the SEER database

The univariate log-rank test showed that, beside of the number of retrieved LNs, other clinicopathological factors, including age more than 60 years, White race, poor/undifferentiated tumor grade, overlapping lesion of stomach, mucinous and signet-ring cancer as well as advanced TN stages were regarded as significant risk factors for 5-year CSS rate (P< 0.001). Multivariate analysis with Cox regression demonstrated that more retrieved LNs exhibited survival advantage (LNs: 7-14, hazard ratio (HR) 0.586; 95% confidence interval [CI] 0.536-0.640; LNs: ≥15, HR 0.390; 95% CI 0.356-0.427) (P< 0.001) (Table 3).

### Prognostic nomogram for CSS and OS

**Figure 3:**
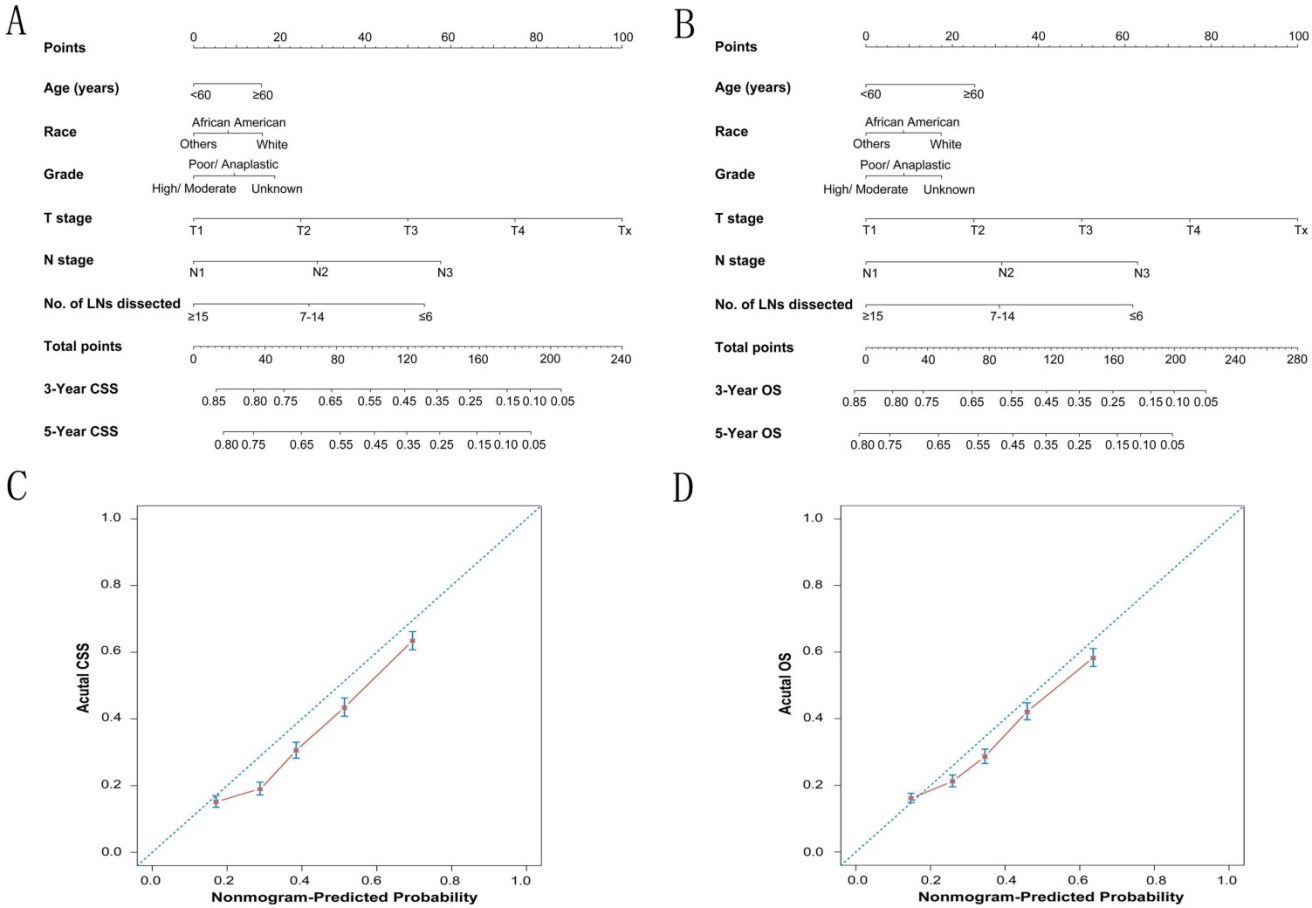
** The calibration plots for predicting CSS and OS of gastric cancer patients.** Nomograms can be interpreted by summing up the points assigned to each variable, which is indicated at the top of scale. The total points can be converted to predicted 3-year and 5-year probability of survival for a patient in the lowest scale (A, B). The Harrell's c-indexes for CSS and OS prediction were 0.663 (95% CI: 0.655-0.671) and 0.654 (95% CI: 0.646-0.662) (P< 0.001), respectively. Calibration curves for 5-year CSS (C) and 5-year OS (D) using nomograms with clinicopathological characteristics and LN counts are shown. The x-axis is nomogram-predicted probability of survival and y-axis is actual survival. The reference line is 45°and indicates perfect calibration.

To predict CSS and OS in GC patients, the external validation of nomograms was performed and predictive factors were determined by cox stepwise regression analysis (Fig. 3A and 3B) 9. Each variable was assigned a score at the top of scale. By counting the total score, we were able to draw a straight line down to predict 3-year and 5-year probability of survival for a patient at each time point. The Harrell's c-indexes to predict CSS and OS prediction were 0.663 (95% CI: 0.655-0.671) and 0.654 (95% CI: 0.646-0.662) (P< 0.001), which were significantly higher than those of the model without the variable of dissected LNs (CSS: 0.663 versus 0.64; OS: 0.654 versus 0.63) (P< 0.001). Calibration curves for two nomograms (Fig. 3C and 3D) revealed no deviations from the reference line and no need of recalibration. The decision curve analysis indicated that for most of the threshold probabilities for 5-year CSS and OS, with LN count nomogram achieved a greater net benefit compared with without LN count (Fig. S1).

### Subgroup analysis of retrieved LNs effect on GCSS according to pN categories

We then further analyzed the effect of retrieved LNs on GCSS rates in each stage. After stratifying by the confounding factors, the univariate analysis of retrieved LNs effect on GCSS rates showed that the retrieved LNs exhibited increased 5-year GCSS rates across several N subgroups (P< 0.001). Comparing with the patients who had ≤6 retrieved LNs, there was a 35.0% and 27.1% improvement in 5-year GCSS in those ≥15 retrieved LNs patients in N1 and N2 stage, and stills a 7.5% improvement when compared with 7-14 retrieved LNs patients in N3 stage (P< 0.001). Besides, the retrieved LNs were also validated as an independent predictor of survival in multivariate Cox regression in N1 stage (LNs≥15, HR 0.373, 95% CI 0.325-0.427, P< 0.001), N2 stage (LNs≥15, HR 0.406, 95% CI 0.352-0.469, P< 0.001) and N3 stage (LNs≥15, HR 0.789, 95% CI 0.719-0.865, P< 0.001) (Table 4).

## Discussion

Although the increased trend in the diagnosis of GC, the prognosis of GC is still poor and the 5-year survival was less than 30% 10. Radical gastrectomy is considered as the only potentially curative therapy for all the GC patients 11. LN metastases in gastric cancer are well recognized as one of the most important prognostic factors, and regional lymph nodes dissection could improve the long-term survival 12, 13. The American Joint Committee on Cancer (AJCC) has recommended a minimum of 15 lymph nodes should be examined in order to get accurate postoperative stage 14, 15. According to the 8th edition TNM classification, the minimum examined lymph node count is not mandatory for proper staging, although more than 16 examined LNs has been proposed to ensure the accurate prognosis of pN stage since 2009^16^. Moreover, the number of retrieved LNs has been confirmed as an independent prognosis factor in esophageal cancer 17, colon cancer 18 et al. However, debate also exists regarding the importance and the number of retrieved LNs in gastric cancer. Okajima et al. suggested that 25 or more LN harvests might be sufficient for nodal staging 19. Liu et al. recommended no less than 15 total LNs should be pathologically examined in patients with N1-3 20. Shi et al. also reported that negative lymph node counts, which did not take positive LN into consideration, could predict prognosis for patients with gastric cancer 5. In addition, in node-negative gastric cancer, Zheng et al. found retrieved LN counts was associated with long-time survival outcomes. The higher the LN count, the better the survival would be 8. Deng et al. found that more than 15 examined LNs in node-negative GC patients were mandatory for improvement in the prognostic assessment accuracy 21-23. However, the relationship between total LN counts and GCSS has not been fully investigated in a large population.

According to all present clinical guidelines, total LN counts for gastric cancer are the main concern. In view of the importance of total LN counts, in this study, we mainly investigate the prognostic value of total LN counts in node-positive GC. We first used the X-tile program to divided GC patients into low, middle, and high-risk groups, and identified 4 and 14 as the optimal cutoff value in terms of GCSS. Then the result was further confirmed in an additional one-by-one cutoff value analysis from 2 to 20. The 5-year GCSS of patients with N or more nodes increased gradually when N reached 14. After the number 15, the survival rates were roughly stable between the compared groups. Above results indicated that inadequate LN harvest in node-positive gastric cancer patients may reflect limited lymph node dissection for gastric cancer, which increased the risk of recurrence and metastasis. Besides, we also validated retrieved LN counts as an independent prognostic factor in node-positive gastric cancer. The survival rates were positively correlated with the number of retrieved LN counts.

The nomogram is a simple statistics-based tool that provides the overall probability of a specific clinical event. For many cancers, nomograms are validated to be more accurate in predicting the probability of an event, such as death or recurrence, when compared with the traditional TNM staging systems 24. The X-tile software is a comprehensive method, based on traditional statistical tests, and yet intuitive for the oncologist. The X-tile plot illustrates the presence of substantial subpopulations and shows the robustness of the relationship between a biomarker and outcome by construction of a two dimensional projection of every possible sub-population 25. In this study, we used nomograms incorporating different retrieved LN number that identified the optimal cut-off value by X-tile program in a large population, and exhibited better predictive accuracy than that of the model without the variable of dissected LNs.

Several hypotheses may explain this finding for the relationship between the number of retrieved LNs and survival. First, total LN counts indicate the actual harvested LNs number intraoperatively. Moreover, it also reflects the properly identified and examined LNs during pathologic analysis of the surgical specimen, which result in cancer upstaging. Second, previous studies have shown that patients with lymphocytic infiltration have a better survival than those who have no infiltration 26, 27. More dissected LNs which are associated with LN counts may reflect a higher host lymphocytic reaction to the tumor 28, 29. Furthermore, we have to remain aware of the fact that increased number of retrieved LNs may attribute to improved surgical techniques. Theoretically, it also reflects an authoritative surgical curability and quality of surgical care or pathology, thus prolonging the survival and disease-free period.

Although this study is based on a large population, there are still potential limitations. First, several important pieces of information regarding surgical options (eg, palliative therapy, radical resection), as well as cancer treatment (chemotherapy, radiotherapy), are not included in the SEER database, which could not be adjusted by our analyses. Second, SEER database also lacks the situation of postoperative adjuvant chemotherapy, and information about the depth of tumor invasion (T4a/T4b), as well as the information of pathology-specific covariates including perineural invasion and vascular invasion which are essential for prognosis evaluation. Third, the number of lymph nodes harvested depends on the quality of surgery and pathology. These variables that cannot be adjusted may differ in different institutions. Despite these limitations, our analysis of the SEER database revealed that total LN counts were an independent prognostic predictor with surgically treated gastric cancer. Increased retrieved LNs count was associated with long-time survival outcomes in node-positive gastric cancer; it could provide more accurate prognostic information than the current node stage system.

## Supplementary Material

Supplementary figure.

## Figures and Tables

**Figure 2:**
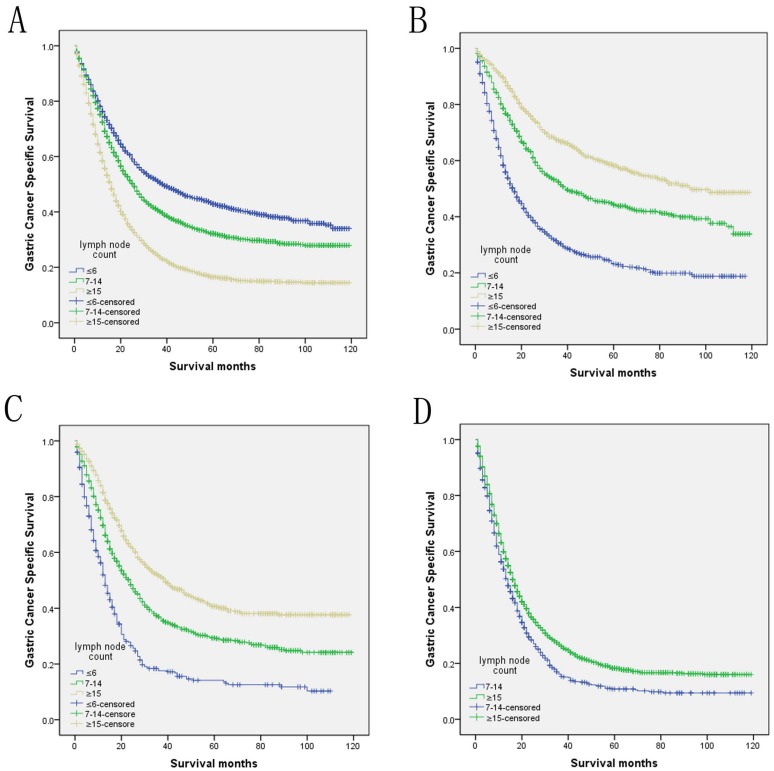
** Log-rank tests of cause specific comparing those who had ≥15, 7-14, and ≤6 positive lymph nodes for** A. all stage: χ2 = 491.935, P < 0.001; B. N1 stage: χ2 = 305.678, P < 0.001; C. N2 stage: χ2 = 200.635, P < 0.001; D. N3 stage: χ2 = 29.113, P < 0.001.

**Table 1:** Demographic and tumor characteristics of patients with node positive gastric cancer

			Subgroup								P value
	All Patients		N1		N2		N3a		N3b		
	n=8475		n=2738		n=2493		n=2252		n=992		
Characteristic	No.	%	No.	%	No.	%	No.	%	No.	%	
**Sex**											0.016
male	5404	63.8	1775	64.8	1613	64.7	1426	63.3	590	59.5	
female	3071	36.2	963	35.2	880	35.3	826	36.7	402	40.5	
**Age**											<0.001
<60	2924	34.5	846	30.9	843	33.8	876	38.9	359	36.2	
≥60	5551	65.5	1892	69.1	1650	66.2	1376	61.1	633	63.8	
**Race**											<0.001
White	5629	66.4	1856	67.8	1681	67.4	1453	64.5	639	64.4	
African American	1170	13.8	381	13.9	364	14.6	301	13.4	124	12.5	
Others	1676	19.8	501	18.3	448	18.0	498	22.1	229	23.1	
**Location**											<0.001
Cardia, NOS	2198	15.9	864	31.6	711	28.5	474	21.0	149	15.0	
Fundus of stomach	255	3.0	76	2.8	92	3.7	56	2.5	31	3.1	
Body of stomach	661	8.0	202	7.4	185	7.4	180	8.0	94	9.5	
Gastric antrum	2118	25.0	652	23.8	631	25.3	596	26.5	239	24.1	
Pylorus	403	4.8	130	4.7	129	5.2	117	5.2	27	2.7	
Lesser curvature of stomach NOS	952	11.2	295	10.8	248	9.9	286	12.7	123	12.4	
Greater curvature of stomach NOS	402	4.7	128	4.7	108	4.3	111	4.9	55	5.5	
Overlapping lesion of stomach	756	8.9	174	6.4	182	7.3	245	10.9	155	15.6	
Stomach, NOS	730	8.6	217	7.9	207	8.3	187	8.3	119	12.0	
**Pathological grading**											<0.001
High/ Moderate	1985	23.4	853	31.2	611	24.5	404	17.9	117	11.8	
Poor/ Anaplastic	6171	72.8	1731	63.2	1813	72.7	1782	79.1	845	85.2	
Unknown	319	3.8	154	5.6	69	2.8	66	2.9	30	3.0	
**Histotype**											<0.001
Adenocarcinoma	5962	70.3	2110	77.1	1825	73.2	1474	65.5	553	55.7	
Mucinous /Signet ring cell	2513	29.7	628	22.9	668	26.8	778	34.5	439	44.3	
**pT stage**											<0.001
T1	581	6.9	373	13.6	159	6.4	41	1.8	8	0.8	
T2	786	9.3	412	15.0	227	9.1	122	5.4	25	2.5	
T3	3596	42.4	1090	39.8	1169	46.9	1007	44.7	330	33.3	
T4	3460	40.8	820	29.9	934	37.5	1078	47.9	628	63.3	
Tx	52	0.6	43	1.6	4	0.2	4	0.2	1	0.1	
**No. of LNs dissected**	17.89(9-23)		13.18(5-18)		15.91(8-20)		19.67(13-24)		31.87(22-38)		<0.001
No. of positive LNs	7.19(2-10)		1.40(1-2)		4.26(3-5)		10.14(8-12)		23.80(18-27)		<0.001

**Table 2:** Univariate analysis for the influence of different cutoffs on GCSS in gastric cancer.

Cutoff	No.	5-year GCCS	Log-rank χ^2^	P value
<2	268	10.2%	125.990	<0.001
≥2	8207	30.3%		
<3	413	13.3%	114.568	<0.001
≥3	8062	30.5%		
<4	591	15.2%	130.281	<0.001
≥4	7884	30.7%		
<5	787	18.7%	99.070	<0.001
≥5	7688	30.8%		
<6	998	18.2%	132.053	<0.001
≥6	7477	31.2%		
<7	1252	20.3%	118.442	<0.001
≥7	7223	31.3%		
<8	1544	21.7%	114.520	<0.001
≥8	6931	31.4%		
<9	1808	22.1%	121.692	<0.001
≥9	6667	31.7%		
<10	2165	23.0%	117.830	<0.001
≥10	6310	31.9%		
<11	2521	24.7%	83.525	<0.001
≥11	5954	31.7%		
<12	2883	24.8%	88.381	<0.001
≥12	5592	32.2%		
<13	3234	25.6%	77.568	<0.001
≥13	5241	32.2%		
<14	3587	25.8%	77.682	<0.001
≥14	4888	32.5%		
<15	3882	26.2%	70.925	<0.001
≥15	4593	32.6%		
<16	4239	26.6%	60.232	<0.001
≥16	4236	32.7%		
<17	4557	26.9%	53.464	<0.001
≥17	3918	32.9%		
<18	4873	26.8%	59.287	<0.001
≥18	3602	33.5%		
<19	5178	27.7%	42.607	<0.001
≥19	3297	32.8%		
<20	5431	28.0%	38.271	<0.001
≥20	3044	32.7%		

**Table 3:** Univariate and multivariate survival analyses for evaluating the influence of the number of retrieved LNs influencing GCSS in node positive gastric cancer patients.

		Univariate analysis		Multivariate analysis	
Variable	5-year GCCS	Log rank χ^2^ test	P	HR(95%CI)	P
Sex		3.059	0.08		
Male	30.4%				
Female	28.5%				
Age		23.923	<0.001		<0.001
<60	32.0%			Reference	
≥60	28.4%			1.295(1.221-1.373)	
Race		51.463	<0.001		<0.001
White	27.6%			Reference	
African American	28.2%			1.042(0.960-1.131)	
Others	37.5%			0.809(0.751-0.871)	
**Location**		72.114	<0.001		0.0251
Cardia, NOS	26.1%			Reference	
Fundus of stomach	31.4%			0.827(0.700-0.978)	
Body of stomach	30.5%			0.769(0.687-0.861)	
Gastric antrum	32.6%			0.778(0.718-0.842)	
Pylorus	29.7%			0.857(0.748-0.982)	
Lesser curvature of stomach NOS	37.1%			0.693(0.625-0.768)	
Greater curvature of stomach NOS	31.2%			0.812(0.707-0.931)	
Overlapping lesion of stomach	23.3%			0.860(0.774-0.955)	
Stomach, NOS	26.6%			0.891(0.801-0.992)	
**Pathological grading**		98.930	<0.001		<0.001
High/ Moderate	37.9%			Reference	
Poor/ Anaplastic	27.4%			1.156(1.077-1.241)	
Unknown	21.7%			1.186(1.021-1.377)	
Histotype		45.168	<0.001		0.060
Adenocarcinoma	32.1%			Reference	
Mucinous/signet ring cell	24.0%			1.061(0.998-1.129)	
pT Stage		731.610	<0.001		<0.001
T1	60.2%			Reference	
T2	51.1%			1.156(0.969-1.380)	
T3	31.7%			1.835(1.584-2.126)	
T4	17.9%			2.604(2.246-3.019)	
Tx	4.2%			4.453(3.216-6.164)	
pN Stage		620.680	<0.001		<0.001
N1	42.9%			Reference	
N2	32.2%			1.371(1.270-1.480)	
N3a	19.7%			2.186(2.010-2.378)	
N3b	9.2%			3.524(3.169-2.919)	
No. of LNs		135.822	<0.001		<0.001
≤6	20.3%			Reference	
7-14	29.0%			0.586(0.536-0.640)	
≥15	32.6%			0.390(0.356-0.427)	

NI: not included in the multivariate survival analysis. P values were adjusted for age, race, location, pathological grading, histotype stage, tumor stage and No. of LNs as covariates.

**Table 4:** Univariate and multivariate survival analyses evaluating the number of retrieved LNs influencing GCSS based on different cancer stage.

		Univariate analysis		Multivariate analysis	
Variable	5-year GCCS	Log rank χ^2^ test	P	HR(95%CI)	P
**pN Stage**					
**N1**		305.678	<0.001		<0.001
No. of LNs					
≤6	23.1%			Reference	
7-14	44.3%			0.581(0.511-0.661)	
≥15	58.1%			0.373(0.325-0.427)	
**N2**		200.635	<0.001		<0.001
No. of LNs					
≤6	13.6%			Reference	
7-14	29.2%			0.582(0.507-0.669)	
≥15	40.7%			0.406(0.352-0.469)	
**N3**		29.113	<0.001		<0.001
No. of LNs					
≤6					
7-14	10.8%			Reference	
≥15	18.3%			0.789(0.719-0.865)	

P values were adjusted for age, race, location, pathological grading, histotype stage and tumor stage as covariates. NI: not included in the multivariate survival analysis.

## References

[1] Jemal A, Bray F, Center MM, Ferlay J, Ward E, Forman D (2011). Global cancer statistics. CA Cancer J Clin.

[2] Wroblewski LE, Peek RM Jr, Wilson KT (2010). Helicobacter pylori and gastric cancer: factors that modulate disease risk. Clin Microbiol Rev.

[3] Torres J, Correa P, Ferreccio C, Hernandez-Suarez G, Herrero R, Cavazza-Porro M (2013). Gastric cancer incidence and mortality is associated with altitude in the mountainous regions of Pacific Latin America. Cancer Causes Control.

[4] Choi KH, Kim BS, Oh ST, Yook JH (2016). Comparison the sixth and seventh editions of the AJCC staging system for T1 gastric cancer: a long-term follow-up study of 2124 patients. Gastric Cancer.

[5] Shi RL, Chen Q, Ding JB, Yang Z, Pan G, Jiang D (2016). Increased number of negative lymph nodes is associated with improved survival outcome in node positive gastric cancer following radical gastrectomy. Oncotarget.

[6] Schwarz RE, Smith DD (2005). Extended lymph node dissection for gastric cancer: who may benefit? Final results of the randomized Dutch gastric cancer group trial. J Clin Oncol.

[7] Smith DD, Schwarz RR, Schwarz RE (2005). Impact of total lymph node count on staging and survival after gastrectomy for gastric cancer: data from a large US-population database. J Clin Oncol.

[8] Zheng WF, Ji TT, Lin Y, Li RZ (2016). The prognostic value of lymph nodes count on survival of patients with node-negative gastric cancer. Oncotarget.

[9] Wang M, Bai J, Tan Y, Wang S, Tian Y, Gong W (2011). Genetic variant in PSCA predicts survival of diffuse-type gastric cancer in a Chinese population. Int J Cancer.

[10] DeSantis CE, Lin CC, Mariotto AB, Siegel RL, Stein KD, Kramer JL (2014). Cancer treatment and survivorship statistics, 2014. CA Cancer J Clin.

[11] Jiang L, Yang KH, Guan QL, Zhao P, Chen Y, Tian JH (2013). Survival and recurrence free benefits with different lymphadenectomy for resectable gastric cancer: a meta-analysis. J Surg Oncol.

[12] Saito H, Fukumoto Y, Osaki T, Fukuda K, Tatebe S, Tsujitani S (2007). Prognostic significance of level and number of lymph node metastases in patients with gastric cancer. Ann Surg Oncol.

[13] Zhou R, Wu Z, Zhang J, Wang H, Su Y, Huang N (2016). Clinical significance of accurate identification of lymph node status in distant metastatic gastric cancer. Oncotarget.

[14] Washington K (2010). 7th edition of the AJCC cancer staging manual: stomach. Ann Surg Oncol.

[15] Cuschieri SA, Hanna GB (2014). Meta-analysis of D1 versus D2 gastrectomy for gastric adenocarcinoma: let us move on to another era. Ann Surg.

[16] Deng J, Liu J, Wang W, Sun Z, Wang Z, Zhou Z (2018). Validation of clinical significance of examined lymph node count for accurate prognostic evaluation of gastric cancer for the eighth edition of the American Joint Committee on Cancer (AJCC) TNM staging system. Chin J Cancer Res.

[17] Hsu PK, Huang CS, Wang BY, Wu YC, Chou TY, Hsu WH (2013). The prognostic value of the number of negative lymph nodes in esophageal cancer patients after transthoracic resection. Ann Thorac Surg.

[18] Lin XJ, Yu N, Lin XG, Zhang YF, Chen Y, Zhang K (2016). A clinical survey of pain in Parkinson's disease in Eastern China. International psychogeriatrics.

[19] Okajima W, Komatsu S, Ichikawa D, Kosuga T, Kubota T, Okamoto K (2016). Prognostic impact of the number of retrieved lymph nodes in patients with gastric cancer. J Gastroenterol Hepatol.

[20] Liu C, Lu Y, Jun Z, Zhang R, Yao F, Lu P (2009). Impact of total retrieved lymph nodes on staging and survival of patients with gastric cancer invading the subserosa. Surg Oncol.

[21] Deng J, Yamashita H, Seto Y, Liang H (2017). Increasing the Number of Examined Lymph Nodes is a Prerequisite for Improvement in the Accurate Evaluation of Overall Survival of Node-Negative Gastric Cancer Patients. Ann Surg Oncol.

[22] Chu X, Yang ZF (2015). Impact on survival of the number of lymph nodes resected in patients with lymph node-negative gastric cancer. World J Surg Oncol.

[23] Xu D, Huang Y, Geng Q, Guan Y, Li Y, Wang W (2012). Effect of lymph node number on survival of patients with lymph node-negative gastric cancer according to the 7th edition UICC TNM system. PLoS One.

[24] Sternberg CN (2006). Are nomograms better than currently available stage groupings for bladder cancer?. J Clin Oncol.

[25] Camp RL, Dolled-Filhart M, Rimm DL (2004). X-tile: a new bio-informatics tool for biomarker assessment and outcome-based cut-point optimization. Clinical cancer research: an official journal of the American Association for Cancer Research.

[26] Fernandez-Acenero MJ, Galindo-Gallego M, Sanz J, Aljama A (2000). Prognostic influence of tumor-associated eosinophilic infiltrate in colorectal carcinoma. Cancer.

[27] Canna K, McArdle PA, McMillan DC, McNicol AM, Smith GW, McKee RF (2005). The relationship between tumour T-lymphocyte infiltration, the systemic inflammatory response and survival in patients undergoing curative resection for colorectal cancer. Br J Cancer.

[28] George S, Primrose J, Talbot R, Smith J, Mullee M, Bailey D (2006). Will Rogers revisited: prospective observational study of survival of 3592 patients with colorectal cancer according to number of nodes examined by pathologists. Br J Cancer.

[29] Morris M, Platell C, Iacopetta B (2008). Tumor-infiltrating lymphocytes and perforation in colon cancer predict positive response to 5-fluorouracil chemotherapy. Clin Cancer Res.

