# Supplementary material for: Effect of lymph nodes count in node-positive gastric cancer
Source: J Cancer. 2019 Sep 7;10(23):5646–53. doi: 10.7150/jca.30979 (PMC6843880; doi:10.7150/jca.30979)

Fig S1. Decision curve analyses of the 5-year CSS (A) and OS (B) predictions. The dashed lines indicate the net benefit of using the nomograms based on with lymph node count (solid full line) and without lymph node count (solid full line). The assumption that all of the patients will die is shown with a grey line, and the assumption that all of the patients will survive is indicated with a black line. Threshold probability = 1 - predicted 5-year CSS and OS.

A

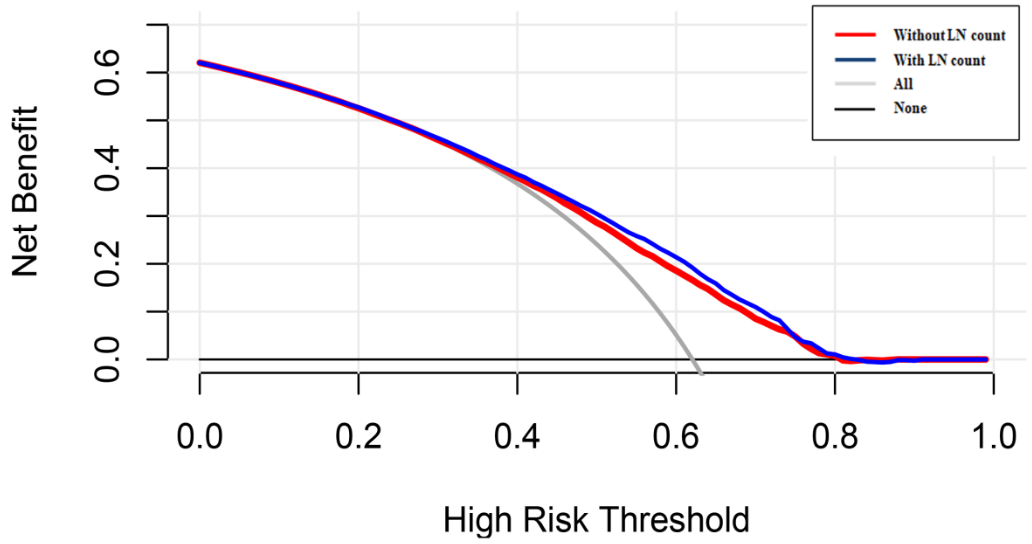

B

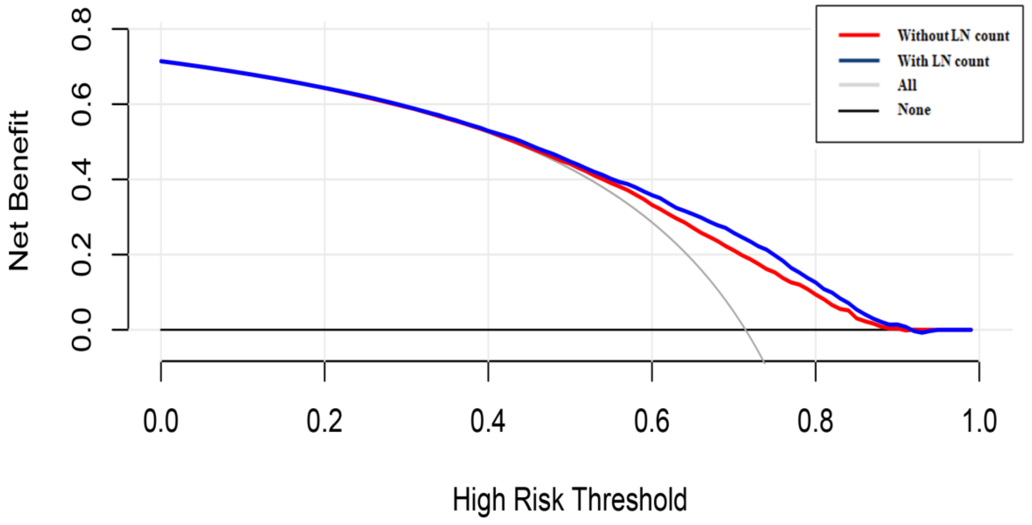

Supplement: Supplementary file 1 — Supplementary figure. [file jcav10p5646s1.pdf]
